# Neonatal adrenal hemorrhage and neonatal jaundice

**DOI:** 10.4103/0971-9261.70650

**Published:** 2010

**Authors:** Viroj Wiwanitkit

**Affiliations:** Wiwanitkit House, Bangkhae, Bangkok, Thailand - 10160, Bangkok

Sir,

I read the recent publication on neonatal adrenal hemorrhage by Qureshi *et al*. with great interest.[1] The authors demonstrated that neonatal adrenal hemorrhage could present with late-onset neonatal jaundice. Indeed, neonatal adrenal hemorrhage is a rare neonatal disorder, which might cause early neonatal jaundice and neonatal anemia (due to extravascular blood loss). The observation on the late-onset neonatal jaundice in this case is an interesting point to be discussed.[2] Qureshi *et al*. concluded that this observation was due to neonatal adrenal hemorrhage.[2] There might be other possible coexisting causes of late-onset neonatal jaundice (such as breast milk jaundice, sepsis, conjugation defect, thyroid and metabolic disorder, etc.) in this case that is not completely investigated.[3]
